# mRNA-LNP Vaccines Targeting SmpA-PLD and OmpK-Omp22 Induce Protective Immunity Against *Acinetobacter baumannii*

**DOI:** 10.3390/vaccines13070764

**Published:** 2025-07-19

**Authors:** Cong Liu, Xingyun Wang, Yueling Zheng, Xingyue Gao, Jiahui Jin, Xing Cheng, Yunjiao He, Peng George Wang

**Affiliations:** 1 Department of Pharmacology, School of Medicine, Southern University of Science and Technology, Shenzhen 518000, China; 11930759@mail.sustech.edu.cn (C.L.); 12331439@mail.sustech.edu.cn (X.W.); 12332989@mail.sustech.edu.cn (Y.Z.); 12233051@mail.sustech.edu.cn (X.G.); 12233050@mail.sustech.edu.cn (J.J.); 2 Department of Materials Science and Engineering, Southern University of Science and Technology, Shenzhen 518000, China; chengx@sustech.edu.cn

**Keywords:** mRNA vaccine, *Acinetobacter baumannii*, LNP, bacterial infection

## Abstract

Background: *Acinetobacter baumannii* (*A. baumannii*) has emerged as a critical human pathogen, causing high mortality rates among hospitalized patients and frequently triggering nosocomial outbreaks. The increasing prevalence of multidrug-resistant (MDR) *A. baumannii* poses a pressing threat to public health. To date, no commercially available vaccine against *A. baumannii* has been developed for clinical use. messenger RNA (mRNA)–lipid nanoparticle (LNP) vaccines have emerged as a promising vaccination strategy. Methods: In this work, we developed two mRNA vaccines targeting SmpA-PLD and the fusion protein of outer membrane proteins OmpK and Omp22. The mRNA was encapsulated in LNP and administered to BALB/c mice. We evaluated humoral and cellular immune responses, bacterial burden, inflammation, and protective efficacy against *A. baumannii* infection in a sepsis model. Results: These mRNA vaccines triggered robust humoral and cellular immune responses in BALB/c mice, reduced bacterial burden and inflammation in sepsis models, and provided significant protection against *A. baumannii* infection. Notably, the OmpK-Omp22 vaccine exhibited superior protective efficacy, reducing bacterial loads in various organs and improving survival rates in the sepsis model compared to the SmpA-PLD vaccine. Conclusions: Our findings demonstrate mRNA-LNP vaccine technology as a versatile and promising platform for the development of innovative therapeutics against *A. baumannii*, with the potential to mitigate acute disease and promote bacterial decolonization. These findings pave the way for the development of urgently needed and effective antibacterial vaccines.

## 1. Introduction

*Acinetobacter baumannii* (*A. baumannii*) is a prevalent pathogen causing nosocomial infections and is commonly associated with multidrug resistance. Infections caused by *A. baumannii* often lead to pneumonia, wound infections, and sepsis, with a mortality rate as high as 50% [1,2]. Patients with underlying diseases or those undergoing surgical procedures are particularly susceptible [3]. Antibiotic therapy has enabled the successful treatment of *A. baumannii* infections. Nonetheless, the emergence and spread of multidrug-resistant (MDR) *A. baumannii* has been highlighted as a global health crisis [4]. The Antimicrobial Resistance Surveillance System (https://www.carss.cn/, accessed on 18 November 2024) revealed that the resistance rate of *A. baumannii* to carbapenems averaged 56.1% [5]. The World Health Organization has designated *A. baumannii* as a critical research priority; thus, there is an urgent need for new strategies to combat *A. baumannii* infection [6].

Vaccines are the most scientific, economical, and effective means of preventing infectious diseases [7]. Vaccines targeting specific antigens trigger the production of antibodies by active immunity, prevent pathogen infections, and reduce the emergence of MDR *A. baumannii* [8,9]. To date, there are no licensed vaccines against *A. baumannii*. Due to the diversity of *A. baumannii*, extensive antigen variation, and strong resistance to most antibiotics, conventional vaccines are more time-consuming and have potential safety concerns. Messenger RNA (mRNA) lipid nanoparticles (LNP) vaccines have been recognized as a next-generation vaccine due to the safety and effectiveness in combating the pandemic of coronavirus disease 2019 (COVID-19) [10,11]. In contrast to conventional vaccines, mRNA vaccine has low-cost manufacturing advantages, high safety potential, high manufacturing efficiency, and rapid development potential, inducing robust humoral and cellular immune responses [12]. mRNA vaccines have shown promising results against viral diseases and cancers [13,14]; however, mRNA vaccines for bacterial pathogens remain largely undeveloped. Therefore, developing mRNA vaccines holds significant promise for combating *A. baumannii* infection.

Outer membrane proteins (Omps) are pivotal in mediating antibiotic resistance and contributing to the pathogenicity of *A. baumannii*. Omp22, a highly conserved outer membrane protein, interacts with peptidoglycan to modulate the biogenesis of outer membrane vesicles. These vesicles serve as key carriers for the secretion of molecules that can either enhance virulence, induce cytotoxicity, or confer resistance to antimicrobial agents [15]. Outer membrane protein K (OmpK) is specific for the nucleoside-forming ion channels [16]. OmpK and Omp22 are capable of eliciting an immune response in the host. These outer membrane proteins on the *A. baumannii* surface are recognizable by the immune system and stimulate the host to produce specific antibodies, thereby conferring immunological protection [16]. Moreover, the small protein A (SmpA) is universally expressed with homology across the genomes of various bacteria. SmpA constitutes one of the essential components of the cellular membrane and is considered a vital structural element in maintaining membrane integrity, playing a significant role in the formation of the outer membrane. Phospholipase D (PLD) has been demonstrated to exert a crucial role in the process of hematogenous dissemination of pathogens, and studies have confirmed that PLD is a virulence factor in *A. baumannii*. SmpA and PLD, as critical virulence factors, facilitate the hematogenous dissemination of *A. baumannii*. Reverse immunology and in silico analysis have indicated that SmpA and PLD hold promise as potential vaccine candidates for the prevention of *A. baumannii* infections [17].

In this study, we report the first mRNA vaccines against *A. baumannii*, and we chose four highly conserved proteins of *A. baumannii* (SmpA, PLD, OmpK, and Omp22) as the candidate antigens. By using our established mRNA-LNP platform [18], we constructed two mRNA vaccines based on the OmpK-Omp22 and SmpA-PLD antigens, evaluated the antigen-specific humoral and cellular responses, and assessed protection effectiveness against a fully virulent *A. baumannii* challenge in mice. The results of this study demonstrated that mRNA vaccines targeting OmpK-Omp22 and SmpA-PLD have the potential to combat *A. baumannii* infections, which paves the way for the development of mRNA vaccines against other bacterial pathogens.

## 2. Materials and Methods

### 2.1. Design and Synthesis of mRNA Vaccine Constructs

The sequences of four *A. baumannii* surface antigens, SmpA, PLD, OmpK, and Omp22, were codon optimized. The sequences for these proteins were sourced from the NCBI database. The DNA sequences encoding the two mRNA vaccines SmpA-PLD and OmpK-Omp22 were synthesized, cloned, and inserted into the pVAX1 plasmid (GENEWIZ, South Plainfield, NJ, USA). A 6×His tag was appended to the C-terminus for detection and purification. To enhance mRNA stability and translation efficiency, we incorporated the 5′ and 3′ untranslated regions (UTRs) from the human beta-globin gene, and a 110-nt poly(A) tail. These synthetic gene fragments were then cloned into the pVAX1 plasmid, yielding the plasmids pVAX1-SmpA-PLD and pVAX1-OmpK-Omp22, which serve as templates for mRNA synthesis. The P2A sequence was used in the mRNA vaccine SmpA-PLD to enable the simultaneous translation of the different *A. baumannii* proteins. The flexible linker (GGGGS)_3_ was used in the mRNA vaccine OmpK-Omp22 to confer optimal structure and stability for our target fusion protein.

### 2.2. In Vitro Transcription and Purification of mRNA

The pVAX1-SmpA-PLD and pVAX1-OmpK-Omp22 plasmids were linearized via the enzyme XhoI (NEB) and used as templates for in vitro mRNA transcription (IVT). IVT was performed via T7 polymerase (NEB) mediated DNA transcription in the presence of a murine RNase inhibitor (NEB), nucleoside triphosphates (NTPs, NEB), CleanCap^®^ Reagent AG (3′ OMe) (Trilink, San Diego, CA, USA), and inorganic pyrophosphatase (NEB). The reaction was allowed to proceed at 37 °C for 2 h, followed by purification of the mRNA using LiCl (Thermo Fisher Scientific, Waltham, MA, USA). Finally, the concentration of the synthesized mRNA was quantified using a NanoDrop 2000c UV spectrophotometer (Thermo Fisher Scientific, Waltham, MA, USA).

### 2.3. mRNA Transfection into HEK293T Cells

HEK293T cells were seeded in 24-well plates at 200,000 cells/well; 16 h later, the 293T cells were transfected with mRNA-SmpA-PLD and mRNA-OmpK-Omp22 using Lipofectamine 2000 (Thermo Fisher Scientific) following the protocol provided by the manufacturer. Briefly, 3 µg mRNA was diluted in 250 µL of Opti-MEM medium (Gibco, New York, NY, USA) and pre-incubated with 3 µL Lipofectamine 2000 (Thermo Fisher Scientific, Waltham, MA, USA) for 10 min at room temperature. This complex was then added to the cells. After an incubation period of 4 to 6 h, the medium was replaced with Opti-MEM medium (Thermo Fisher Scientific, Waltham, MA, USA). Thirty-six hours after transfection, the cells were collected for subsequent protein analysis.

### 2.4. Western Blot

Protein expression in whole-cell lysates from cells transfected with mRNA was evaluated via Western blotting. Samples were denatured with 5× loading buffer by boiling for 10 min, separated by electrophoresis on a 4–20% SurePAGE Bis-Tris gel, and transferred to polyvinylidene fluoride (PVDF) membranes with the eBlot^®^ L1 system (GenScript, Nanjing, China), which offers high-efficiency wet blotting. The membrane was then incubated with a blocking solution consisting of 5% bovine serum albumin (BSA) in 1× phosphate-buffered saline containing 0.1% Tween-20 (PBST). Detection of the SmpA-PLD and OmpK-Omp22 was achieved using an anti-His-HRP antibody at a dilution of 1:10,000 for 2 h, followed by thorough washing with PBST. The immunoreactive bands were visualized using the SHST chemiluminescence imager (SHST, Hangzhou, China).

### 2.5. Formulation of mRNA-LNP

The SmpA-PLD and OmpK-Omp22 mRNA-LNPs were prepared using a microfluidic mixer as described in our previous report [19]. Briefly, the lipid mixture was dissolved in ethanol containing an ionizable cationic lipid (SM102), cholesterol, DSPC, and PEGylated lipid (with a molar ratio of 50:38.5:10:1.5). The mRNA was solubilized in 20 mM citrate buffer (pH 4.0). This mRNA solution was then combined with the lipid mixture at a ratio of 1:3 using a T-mixer (Inano D, Micro&Nano Technology Inc., Shanghai, China) to facilitate the formation of LNPs. Formulations were dialyzed against PBS (pH 7.4) using pre-sterilized Amicon^®^ Ultra-15 centrifugal filters (Millipore, Burlington, NJ, USA) for 16 h to eliminate ethanol. Subsequently, the LNPs were filtered via a 0.22 µm filter to ensure sterility, and the mRNA-LNP was stored at 4 °C.

### 2.6. Particle Size and Encapsulation Efficiency of mRNA-LNP

The particle size of the mRNA-LNP was measured using a Malvern Zetasizer instrument (Malvern, Worcestershire, UK). The encapsulation efficiency of mRNA within the LNPs was quantified by Quant-iT ™ RiboGreen ™ RNA kit (Invitrogen, Carlsbad, CA, USA).

### 2.7. Mice and Strains

Specific pathogen-free female 6–8 weeks old BALB/c mice were purchased from Kangde Biological (Guangzhou, China). The conduct of all animal-based experiments adhered to stringent ethical guidelines and received approval from the Animal Experimentation Ethics Committee of Southern University of Science and Technology. The *A. baumannii* strain ATCC19606 was sourced from the China Culture Collection Center, Beijing, China.

### 2.8. Immunization of Mice and Mouse Sepsis Model

Female BALB/c mice (6 to 8 weeks old) were randomly divided into three groups: mRNA-SmpA-PLD-LNP (*n* = 8), mRNA-OmpK-Omp22-LNP (*n* = 8), and LNP (*n* = 8). BALB/c mice were vaccinated intramuscularly with 15 µg/100 µL SmpA-PLD, 15 µg/100 µL OmpK-Omp22, and LNP. Blank LNP served as the placebo group. Serum was collected at 0, 14, and 28 days, and then stored at −20 °C until the ELISA assays. Spleens were collected 3 weeks after administration of the last booster shot for evaluation of cell-mediated immune responses by flow cytometry and ELISPOT. For bacteremia challenge, the mice were injected intraperitoneally with 5 × 10^7^ CFU of ATCC19606. Survival of infected mice was monitored daily for 14 days.

### 2.9. Enzyme-Linked Immunosorbent Assay

IgG expression levels were quantified using an enzyme-linked immunosorbent assay (ELISA). The 96-well ELISA plates (Corning, New York, NY, USA) were precoated with 2 µg/mL of recombinant SmpA-PLD and OmpK-Omp22 protein and incubated at 4 °C overnight. Non-specific binding was minimized by blocking the plates with 5% bovine serum albumin (BSA) in phosphate-buffered saline with Tween-20 (PBST) at 37 °C for 1 h. After thorough washing with PBST, diluted mouse sera were added and incubated at room temperature for 2 h. Following three additional washes, the plates were incubated with a horseradish peroxidase (HRP)-conjugated anti-mouse IgG secondary antibody at a dilution of 1:10,000 (Proteintech, Rosemont, IL, USA). The colorimetric reaction was developed with TMB substrate (Beyotime, Shanghai, China), and the absorbance was quantified at 450 nm using a Synergy HTX microplate reader (BioTeK, Beijing, China).

### 2.10. Flow Cytometry Analyses

T cell responses specific to the antigen were evaluated using a multicolor flow cytometric analysis. Splenocytes were harvested from both immunized and non-immunized mice, and 2 × 10^6^ cells per sample in 100 µL volumes were incubated with SmpA-PLD or OmpK-Omp22 protein at 37 °C for 4 h to induce stimulation. Brefeldin A (Thermo Scientific) was introduced to the splenocyte cultures and incubated for an additional 6 h. The cells were then washed with PBS containing 0.5% BSA and surface-stained with APC/Fire 750 anti-mouse CD3, FITC anti-mouse CD4, and Brilliant Violet 510 anti-mouse CD8a antibodies (BioLegend, San Diego, CA, USA). Following surface staining, cells were fixed and permeabilized using a Fixation/Permeabilization Solution Kit (BD Biosciences, Franklin Lakes, NJ, USA) and intracellularly stained with PE anti-mouse IFN-γ antibodies (BioLegend). The flow cytometric data were processed and analyzed using FlowJo software (v10.8.1).

### 2.11. Enzyme-Linked Immunospot (ELISPOT) Assay

Fourteen days after the second booster vaccination, mice were euthanized, and their spleens were harvested to prepare single-cell suspensions of splenocytes. Cytokine-specific ELISPOT assays (Dakewe, Shenzhen, China) were performed to analyze IFN-γ-producing splenocytes from both vaccinated and control groups, following the manufacturer’s protocol. Briefly, splenocytes from immunized mice were seeded at a density of 1 × 10^5^ cells/well and stimulated with individual antigens (0.2 μg/well); they were then incubated for 24 h at 37 °C. Following incubation, wells were treated with biotinylated primary monoclonal antibodies and incubated for 1 h at 37 °C, followed by streptavidin–HRP conjugate and color development using TMB solution. The Spots were quantified by an Immunospot analyzer (Mabtech, Nacka, Sweden).

### 2.12. Evaluation of Bacterial Load

Following a 24 h post-infection with a dose of 5 × 10^7^ CFU of ATCC19606, the mice were monitored, euthanized, and their organs were harvested and weighed. Samples from the blood, lungs, liver, spleen, and kidneys were collected, homogenized, and suspended in 1 mL of sterile PBS. These homogenates were then serially diluted in sterile PBS, and 50 µL of each dilution was plated onto solid growth media and incubated at 37 °C overnight. The numbers of CFUs per gram of tissue were subsequently calculated from each plate.

### 2.13. Histopathological Assay

A representative mouse from each group was euthanized 24 h post-infection for histological examination. The lung was fixed with 4% paraformaldehyde and embedded in paraffin. Tissue sections were then stained with hematoxylin and eosin to analyze the tissue morphologies.

### 2.14. Statistical Analysis

Data analysis was performed using SPSS version 15.0 and GraphPad Prism version 8.0. Data are presented as mean ± standard error of the mean (SEM). Statistical significance was evaluated using parametric tests: The Student’s *t*-test for comparisons between two groups and one-way analysis of variance (ANOVA) for assessments involving more than three groups. Post hoc pairwise comparisons among multiple groups were conducted with Tukey’s honestly significant difference test to adjust *p*-values for multiple comparisons. Prior to parametric analyses, the normality of data distribution was assessed using the Shapiro-Wilk test, and all datasets included in t-tests and ANOVA satisfied the normality assumption. Significance was set at *p* < 0.05, with the following notation: * *p* < 0.05, ** *p* < 0.01, *** *p* < 0.001, **** *p* < 0.0001, and ns for non-significance. For pairwise comparisons, a two-tailed Student’s *t*-test was employed. Survival data were analyzed using the Wilcoxon log-rank test, with significance indicated at ** *p* < 0.01.

## 3. Results

### 3.1. Design of mRNA Vaccines Against A. baumannii

**Figure 1:**
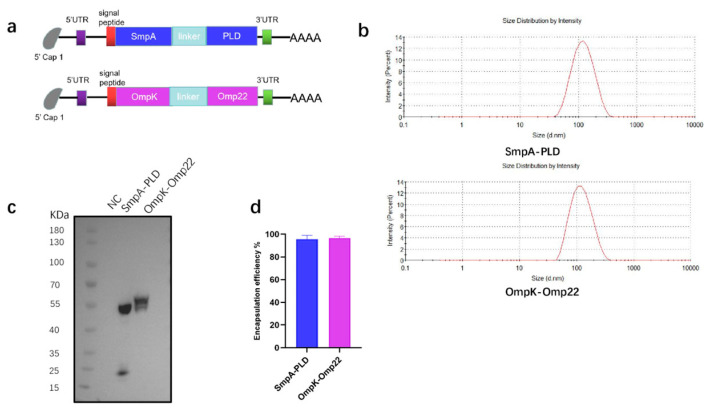
Preparation and characterization of mRNA-SmpA-PLD and mRNA Ompk-Omp22 vaccine. (**a**) The construct of mRNA-SmpA-PLD and mRNA Ompk-Omp22 vaccine for *A. baumannii*. (**b**) The size distribution of the LNPs was measured by a Malvern particle size instrument. (**c**) The expression of mRNA-SmpA-PLD and mRNA-Ompk-Omp22 in 293T cells was analyzed by Western blot. (**d**) The encapsulation efficiency of the LNPs was determined by the Ribogreen assay.

To address the urgent need for broad-spectrum vaccine candidates against *A. baumannii*, four immunogenic antigens-SmpA, PLD, OmpK, and Omp22-were selected as targets for the mRNA coding sequence. The first mRNA vaccine was designed to encode the full-length sequence of the SmpA and PLD genes. Following codon optimization, the coding sequences of SmpA and PLD were linked in tandem via a P2A self-cleaving sequence to enable co-expression of both proteins. The second mRNA vaccine was designed to encode a fusion protein of outer membrane proteins OmpK and Omp22 (Figure 1a). A flexible linker, (GGGGS)_3_, was incorporated for the fusion of the two proteins, aiming to confer the structural flexibility and stability of the fused proteins. The transfection of these mRNA into HEK293T cells was performed to confirm the mRNA translation abilities (Figure 1c). Then the mRNA was encapsulated within LNP through a microfluidic mixer. The particle size of the LNP, which was measured by dynamic light scattering, was 102 nm and 110 nm for each mRNA vaccine, respectively (Figure 1b). The encapsulation efficiency, quantified using the Quant-iT RiboGreen RNA assay, was found to be superior to 95% for both mRNA vaccines (Figure 1d).

### 3.2. mRNA Vaccines Induced Robust Humoral Immune Responses in BALB/c Mice

**Figure 2:**
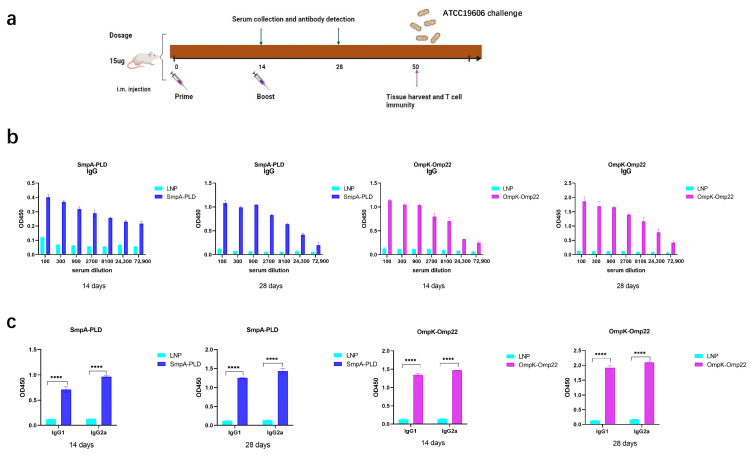
Humoral immune response in mRNA-SmpA-PLD and mRNA Ompk-Omp22 vaccinated mice. (**a**) Schematic diagram of immunization, sample collection, and *A. baumannii* challenge. (**b**) Specific IgG concentrations were assayed by ELISA. (**c**) Specific IgG1 and IgG2a concentrations were assayed by ELISA (**** *p* < 0.0001).

To assess the immunogenicity elicited by the SmpA-PLD and OmpK-Omp22 mRNA vaccines, BALB/c mice (*n* = 8 per group) were intramuscularly immunized twice at 2-week intervals with 15 μg dosage of the respective mRNA vaccines, with a control group receiving empty LNPs (Figure 2a). Serum samples were collected at 14 and 28 days to evaluate antigen-specific humoral responses using enzyme-linked immunosorbent assays (ELISA). The ELISA assays were conducted with plates precoated with recombinant SmpA-PLD or OmpK-Omp22 proteins, which had been heterologously expressed in *Escherichia coli* and subsequently purified. As depicted in Figure 2b,c, the total IgG titers in all mouse groups increased significantly after the boost immunization. Furthermore, the IgG titers in the serum of mice vaccinated with the SmpA-PLD mRNA vaccine were markedly higher than those vaccinated with the OmpK-Omp22mRNA vaccine at all evaluated time points. We further analyzed the IgG subclass induced in each group via ELISA. On day 28 post-initial immunization, both IgG1 and IgG2a were detected at high levels in the serum of mice immunized with either mRNA vaccine. Notably, the IgG1 titer was relatively lower compared to IgG2a, suggesting that immunization with these vaccines elicits a mixed Th1/Th2 immune response with a Th1-biased pattern.

### 3.3. mRNA Vaccines Elicited Strong Cellular Immune Responses in BALB/c Mice

**Figure 3:**
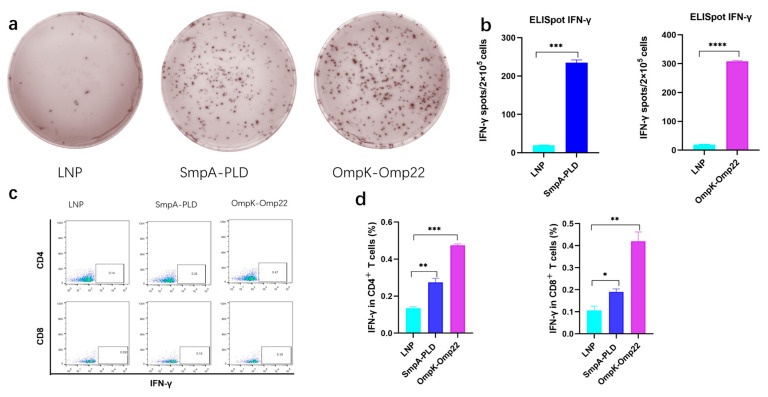
Cellular immune responses in mRNA-SmpA-PLD and mRNA Ompk-Omp22 vaccinated mice. (**a**) Image of the ELISpot assay of IFN-γ-producing T cells. (**b**) The quantitative analysis of IFN-γ-producing T cells. (**c**) The percentages of IFN-γ-producing CD4+ and CD8+ T cells were determined via intracellular cytokine flow cytometry assay. (**d**) The quantitative analysis of IFN-γ-producing CD4+ and CD8+ T cells was determined via intracellular cytokine flow cytometry assay (* *p* < 0.05, ** *p* < 0.01, *** *p* < 0.001, **** *p* < 0.0001).

Vaccine-induced-T cell mediated immune responses play a critical role in combating *A. baumannii* infection [20,21]. To comprehensively assess the immunological outcomes following intramuscular administration of mRNA-SmpA-PLD or mRNA-OmpK-Omp22 in BALB/c mice, two weeks after the second immunization, the splenocytes were isolated for ELISpot and intracellular cytokine flow cytometry assays. ELISpot results demonstrated a significantly higher frequency of IFN-γ-secreting splenocytes in mice immunized with the mRNA-OmpK-Omp22 vaccine compared to those immunized with the mRNA-SmpA-PLD vaccine (Figure 3a,b). Further analysis using flow cytometry revealed that the production of IFN-γ was significantly elevated in CD4+ T cells compared to CD8+ T cells within the mRNA-SmpA-PLD and mRNA-OmpK-Omp22 vaccinated group (Figure 3c).

All these findings revealed that both mRNA-SmpA-PLD and mRNA-OmpK-Omp22 vaccines can induce robust cellular immune responses in immunized mice, with the response induced by the mRNA-OmpK-Omp22 vaccine being more potent than that induced by mRNA-SmpA-PLD. Further investigation is required to elucidate the mechanisms underlying the differential immune responses elicited by these two vaccines.

### 3.4. mRNA Vaccines Provide Potent Protection Against A. baumannii Challenge

**Figure 4:**
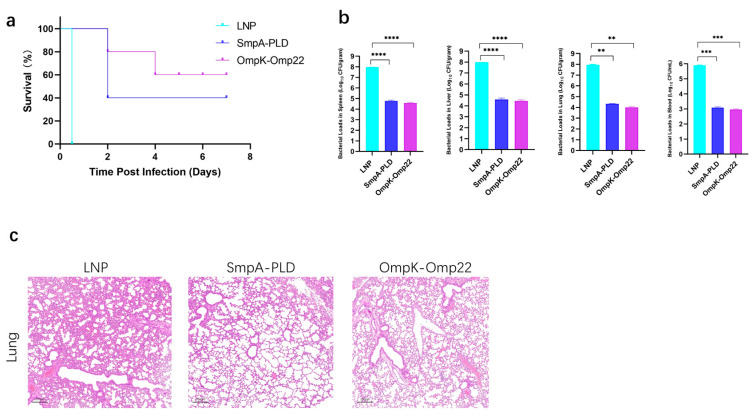
Bacterial load assay and protective effect of mRNA vaccines on the immunized mice. (**a**) Survival curve of mice immunized mRNA vaccine was observed every 12 h for 7 days. (**b**) Evaluation of bacterial load in spleen, liver, lung, and blood of immunized mice groups. (**c**) HE staining of lungs from immunized mice 24 h after infection with ATCC 19606 (** *p* < 0.01, *** *p* < 0.001, **** *p* < 0.0001).

To assess the protective efficacy of developed mRNA vaccine candidates, immunized mice were challenged with 5 × 10^7^ CFU of ATCC19606 through intraperitoneal administration on day 30 after the second immunization (Figure 4a). The survival of mice was recorded in a 14-day period. In the sepsis model, immunization with SmpA-PLD or OmpK-Omp22 significantly increased the survival rate compared to the control mice immunized with empty LNP (Figure 4b). Notably, the OmpK-Omp22 vaccine provided superior protection against ATCC 19606 infection compared to SmpA-PLD, with the survival rate of 60% and 40%, respectively. Additionally, the bacterial load in different organs (lung, liver, spleen, and blood) was quantified 24 h post-challenge (Figure 4b). Immunization with SmpA-PLD or OmpK-Omp22 significantly decreased the bacterial load compared with that in the control mice immunized with LNP. These results demonstrated that immunization with SmpA-PLD or OmpK-Omp22 could simultaneously reduce bacterial colonization and systemic transmission in organs, with OmpK-Omp22 showing a more pronounced effect than SmpA-PLD. Histological analysis of lung tissues from immunized mice challenged with ATCC 19606 at 24 h was performed using H&E staining. As shown in Figure 4c, neutrophil infiltration, alveolar hemorrhage, edema, and necrotic alveoli were observed in the control group. In contrast, all the vaccinated groups substantially reduced lung pathology and inflammatory change, especially in the OmpK-Omp22 group. Remarkably, the OmpK-Omp22 immunization conferred better protection, with lung tissues appearing nearly normal, displaying only minimal hyperaemia and very low levels of inflammatory cells in the interstitium (Figure 4c).

## 4. Discussion

Multidrug-resistant *A. baumannii* poses a severe threat to public health [22,23]. The reliance on conventional monotherapy with a single antibiotic is no longer sufficient to combat *A. baumannii* infection [24]. Consequently, there is an urgent need for novel therapeutic strategies. Among these potential approaches, the vaccine is considered to be a safe and efficacious strategy. Over the past few decades, extensive research has been conducted on the development of vaccines against *A. baumannii*, with several candidate vaccines demonstrating immunogenicity and protective efficacy [25]. However, due to the complexity in the manufacturing process and the weak immunogenicity of single antigens, vaccine development for *A. baumannii* has been stalled at the early clinical research phase. Therefore, there is a critical need for further optimization and innovation in vaccine design to enhance their immunogenicity and protective effects.

mRNA-LNP technology has demonstrated promising results for developing vaccines against viral infections and cancer [10,26]. The mRNA-LNP platforms offer the advantage of rapidly manufacturing and flexibly responding to different targets. In addition, mRNA vaccine induces robust cellular immune responses compared to conventional vaccines [27,28]. In our previous study, we utilized the mRNA-LNP platform to develop the first mRNA vaccine against Pseudomonas aeruginosa [18]. The results have confirmed the feasibility of this platform.

In this study, we have successfully engineered two mRNA-LNP vaccines targeting specific antigens of *A. baumannii*, namely SmpA-PLD and OmpK-Omp22. The results obtained from our comprehensive in vivo and in vitro experiments have provided valuable insights into the potential of these vaccines.

Our findings demonstrated that both vaccines were capable of eliciting significant humoral and cellular immune responses in BALB/c mice. The induction of high levels of antigen-specific IgG, IgG1, and IgG2a antibodies indicates a robust humoral response, which is crucial for neutralizing antibodies and preventing *A. baumannii* invasion. Notably, the IgG titers in the OmpK-Omp22 group were higher than those in the SmpA-PLD group at most time points, suggesting potential differences in the immunogenicity of the antigens or the efficiency of antibody production. However, the presence of both IgG1 and IgG2a in the sera of vaccinated mice indicates a mixed Th1/Th2 immune response, with a bias towards Th1, which is beneficial for clearing *A. baumannii* infection [29].

In terms of cellular immunity, the ELISpot and flow cytometry analyses revealed that both vaccines could activate T cells, especially CD4+ T cells, which play a central role in coordinating the immune response. The higher frequency of IFN-γ-secreting splenocytes in the OmpK-Omp22 group compared to the SmpA-PLD group suggests that the OmpK-Omp22 antigen may be more potent in stimulating T cell responses.

The protective efficacy of the vaccines was clearly demonstrated in the *A. baumannii*-infected mice. Immunization with either SmpA-PLD or OmpK-Omp22 significantly enhanced the survival rate of mice and reduced the bacterial load in various organs, including the lungs, liver, spleen, and blood. These results indicate that the vaccines can effectively limit bacterial colonization and dissemination, thereby protecting the host from severe infection. Interestingly, the OmpK-Omp22 vaccine exhibited superior protection, as evidenced by the nearly normal histological appearance of the lung tissues in this group. These results suggest that the OmpK-Omp22 may be a more promising candidate for vaccine development, although further studies are needed to elucidate the exact mechanisms underlying its enhanced protection. The study is limited to a single *A. baumannii* strain (ATCC19606), and further testing against multidrug-resistant clinical isolates is needed; the optimal valence (e.g., tri- or tetra-antigen constructs) remains to be evaluated in future work.

To the best of our knowledge, this is the first study to use the mRNA-based vaccine against *A. baumannii* infection. In summary, the present study successfully developed two mRNA vaccines targeting *A. baumannii*, and systematically evaluated their immunogenicity and protective efficacy in a mouse model. The results demonstrated that these mRNA vaccines effectively induced a robust humoral immune response and conferred significant protection against challenge infection with *A. baumannii*. These findings provide valuable insights for the optimization of mRNA vaccines for *A. baumannii* and the development of prophylactic and therapeutic vaccines suitable for clinical application.

## 5. Conclusions

This study reports the first mRNA vaccine against *A. baumannii*. Targeting SmpA-PLD and OmpK-Omp22, the mRNA vaccines induced robust humoral and cellular immune responses in mice, reducing bacterial burden in organs and improving survival in a sepsis model. The findings provide valuable insights for mRNA vaccines against *A. baumannii* and open avenues for urgently needed effective antibacterial vaccines.

## Data Availability

The data presented in this study are available in this article.
